# Left Main Compression by a Giant Aneurysm of the Left Sinus of Valsalva: An Extremely Rare Reason for Myocardial Infarction and Cardiogenic Shock

**DOI:** 10.1155/2015/703646

**Published:** 2015-09-15

**Authors:** Bruno L. R. Faillace, Micheli Z. Galon, Marcos Danillo P. Oliveira, Guy F. A. Prado, Adriano A. M. Truffa, Expedito E. Ribeiro, Pedro A. Lemos

**Affiliations:** Department of Interventional Cardiology, Heart Institute (InCor), University of São Paulo, Avenida Dr. Enéas de Carvalho Aguiar 44, 05403-900 São Paulo, SP, Brazil

## Abstract

Aneurysms of the sinus of Valsalva are very rare and mostly located in the right coronary sinus. They might course with dyspnea, fatigue, and acute coronary syndromes. We present herein an extremely rare case report of a 61-year-old woman diagnosed with external left main coronary compression by a giant aneurysm of the left sinus of Valsalva, which was successfully managed with percutaneous coronary intervention.

## 1. Introduction

Aneurysms of the sinus of Valsalva are very rare and mostly located in the right coronary sinus [1], with common protrusion and rupture to right ventricle and atrium, respectively, and are more related to history of infectious endocarditis (IE) with low mortality rate in stable cases [2].

They might course with dyspnea, fatigue, and acute coronary syndromes (ACS) [3, 4]. Clinically recognized myocardial infarction is uncommon in patients with IE and mostly reported as case or series reports [5].

## 2. Case Report

**Figure 1:**
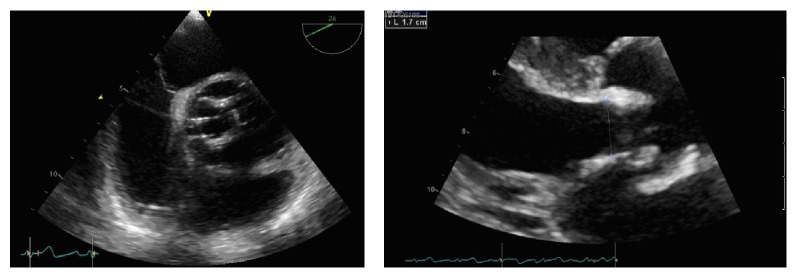
Transesophageal echocardiography images showing thickness of the biological valvar prosthetic leaflets.

A 61-year-old female was submitted four years ago to surgical aortic valve replacement (SAVR) with deployment of a biological valvar prosthesis due to native aortic valve endocarditis. Three years after this procedure, she was admitted to the cardiology emergency department complaining of persistent daily fever and asthenia for the last ten days. Transesophageal echocardiography (TEE) findings (thickness of the prosthetic leaflets, Figure 1) associated with clinical and laboratorial findings (elevated inflammatory markers) were compatible with the Duke criteria [6] for biological aortic prosthesis infectious endocarditis (BAPIE).

**Figure 2:**
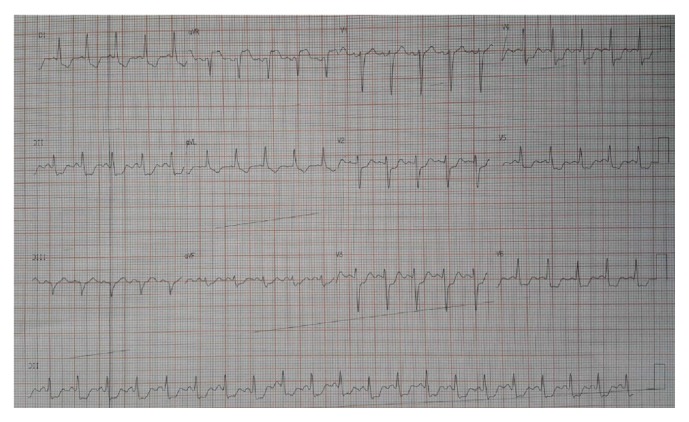
ECG findings: the resting 12 leads electrocardiogram showing the ST segment elevation in the lead aVR with marked diffuse ST segment depression.

**Figure 3:**
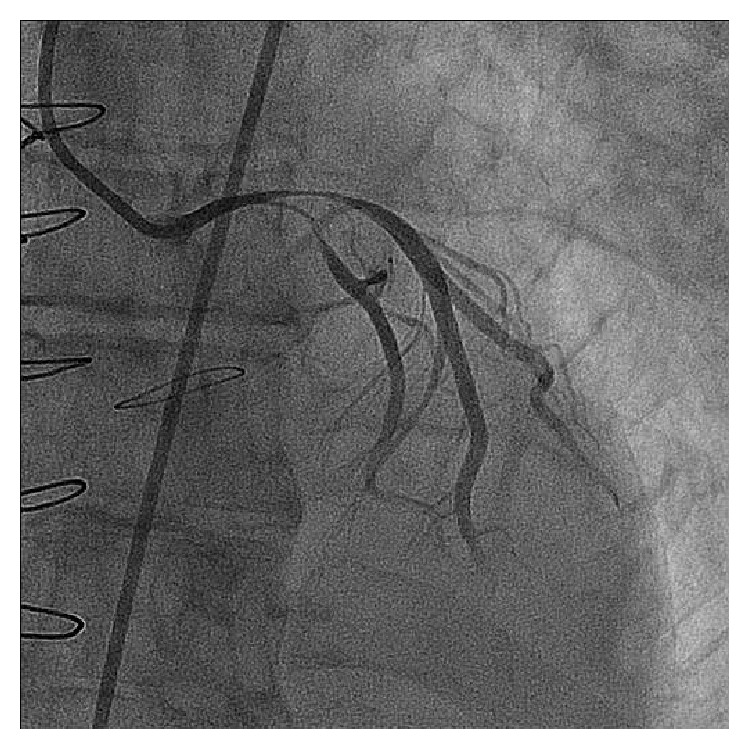
Left coronary artery in RAO caudal projection showing the severe luminal narrowing of the distal LMCA and proximal portions of the LAD and LCx arteries. RAO, right anterior oblique; LMCA, left main coronary artery; LAD, left anterior descending; LCx, left circumflex.

**Figure 4:**
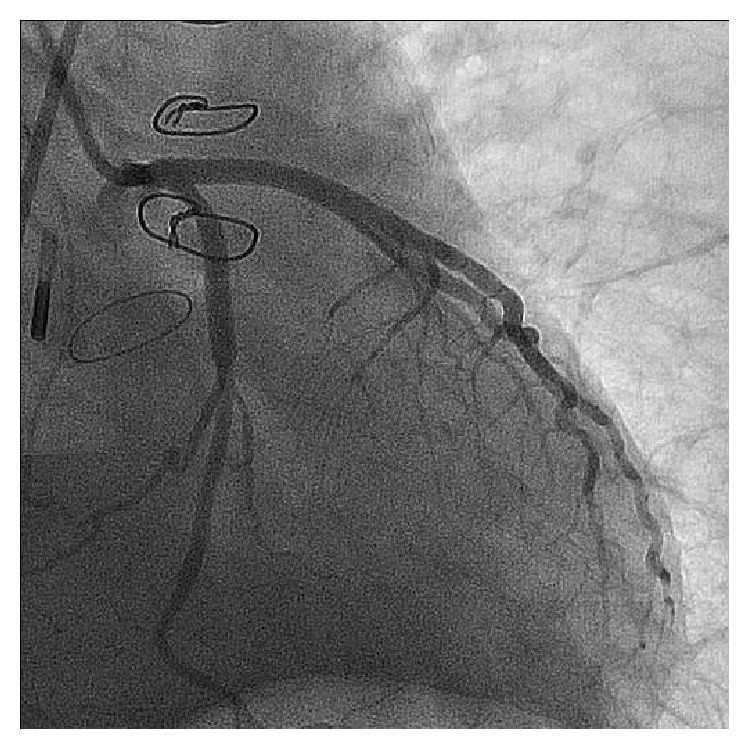
Left coronary artery in RAO caudal projection showing the final result after the PCI of the distal LMCA and proximal portions of the LAD and LCx arteries. RAO, right anterior oblique; PCI, percutaneous coronary intervention; LMCA, left main coronary artery; LAD, left anterior descending; LCx, left circumflex.

During the same hospitalization, twenty days after the antibiotic regimen was introduced, the patient developed hemodynamic instability associated with severe chest pain at rest. The electrocardiogram showed ST segment elevation in the lead aVR and marked diffuse ST segment depression (Figure 2). Emergent coronary angiogram was then performed and evidenced an important luminal narrowing of the distal left main coronary artery (LMCA) and the proximal portions of the left anterior descending (LAD) and left circumflex (LCx) arteries (Figure 3). Due to the refractory hemodynamic compromise, despite the use of high doses of vasopressors and inotropes combined with intra-aortic balloon pumping (IABP) support and invasive mechanical ventilation, it was decided by the percutaneous coronary intervention (PCI). A long metallic stent (3,5 × 28 mm) was then implanted on the LMCA-LAD, which was postdilated with a high pressure noncompliant balloon (4,0 × 15 mm). After that, another long metallic stent (3,5 × 28 mm) was deployed through the struts of the previous stent (according to the “T” and small protrusion technique) into the proximal portion of the LCx. A final simultaneous kissing balloon inflation was then performed. The final angiographic result is showed in Figure 4.

**Figure 5:**
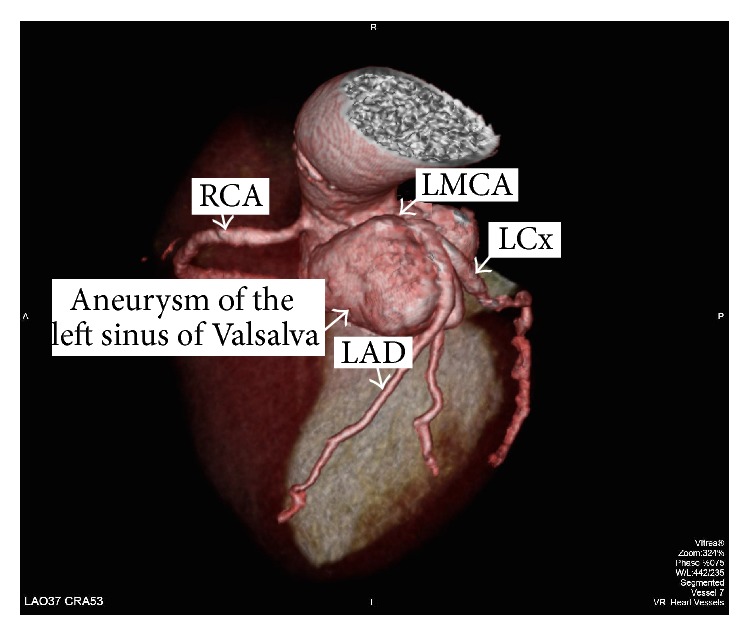
Post-PCI computed tomography angiography showing the giant aneurysm involving the three sinuses of Valsalva and its relationship with the major epicardial coronary arteries. PCI, percutaneous coronary intervention; RCA, right coronary artery; LMCA, left main coronary artery; LAD, left anterior descending; LCx, left circumflex.

The patient was referred to the coronary intensive care unit and the IABP was removed 3 days later. One month after the procedure, a computed tomographic scan angiography (CTSA) was performed, demonstrating the giant aneurysm of the sinuses of Valsalva and its relation to the coronary arteries submitted to the implantation of the metallic stents (Figure 5). Three months later, after a very long-term antibiotic therapy being completed, the patient was discharged home, free of symptoms. One year after the index PCI, she developed new symptoms of advanced heart failure. Thus, a new SAVR was indicated. The patient is, at the time of this report, waiting the call for the surgical procedure.

## 3. Discussion

We present herein a very rare case report of a 61-year-old woman with external LMCA compression by a giant infectious aneurysm of the left sinus of Valsalva, which was successfully managed with PCI.

Large aneurysms of the left sinus of Valsalva can cause protrusion and rupture to the pulmonary artery, left ventricle, myocardium, and epicardium [7]. They often require a surgical approach [8]. In our case, however, the patient presented to us in the catheterization laboratory in an extremely critical status, which led us to proceed with the immediate percutaneous approach.

Leontyev and colleagues [9] reviewed 172 cases of surgically repaired IE complicated by abscess formation and reported a 30-day mortality rate of 25%, with mean one- and five-year survival rates of 55% (±4%) and 50% (±4%), respectively [4]. Of these cases, 76 (44%) involved prosthetic aortic valves, like in our case.

Mansur et al. [10] demonstrated 74% of IE complications in 223 patients, with 100 episodes of cardiac complications (heart failure, valve injury, pericarditis, acute myocardial infarction, conduction abnormalities, fistulous communication, and perivalvular abscess).

Compression of the LMCA causing ST elevation myocardial infarction (STEMI) is very rare [4], but it can cause cardiogenic shock and thus increased mortality.

The management of this condition is always a challenge. The use of vasoactive drugs, mechanical ventilation, and IABP has been described, despite the fact that the last one does not show benefit on long-term mortality in patients with cardiogenic shock [8]. However, in selected individuals, this device can help the management of this disease, with survival improvement.

The European Society of Cardiology guidelines [11] recommend transthoracic echocardiography on any patient suspected of having IE, as well as a follow-up TEE if the transthoracic method is positive or of poor quality, or if the patient has a history of a prosthetic valve or intracardiac device.

Despite the fact that the surgical intervention is not associated with consistent outcomes, missing this diagnosis might be fatal. The exact timing of surgical intervention is controversial and continues to evolve in parallel to advancements in diagnosis and treatment. Due to the urgency in which the patients present during an episode of ACS, it is important to promptly recognize the angiographic features of coronary stenosis secondary to extrinsic compression such as from intracardiac abscesses, masses, or infectious aneurysms of the sinuses of Valsalva, like that in the case reported here.
